# Exploring the role and mechanisms of environmental serious games in promoting pro-environmental decision-making: a focused literature review and future research agenda

**DOI:** 10.3389/fpsyg.2024.1455005

**Published:** 2024-09-16

**Authors:** Jinsong Chen, Miao He, Jing Chen, Chugong Zhang

**Affiliations:** ^1^School of Business Administration, Guizhou University of Finance and Economics, Guiyang, China; ^2^Sichuan Primary and Secondary School Safety Education and Management Research Center, Chengdu Normal University, Chengdu, China; ^3^School of Literature and Journalism, Chengdu Normal University, Chengdu, China; ^4^Institute of Science Innovation and Culture, Rajamangala University of Technology Krungthep, Rajamangala, Thailand

**Keywords:** environmental protection, serious game, gamification, pro-environmental decision-making, pro-environmental behavior

## Abstract

Environmental serious games aim to heighten players’ awareness and comprehension of environmental issues, thus fostering pro-environmental decision-making. Research to date has affirmed these games’ effectiveness in enhancing environmental knowledge and abilities, elevating consciousness regarding environmental matters, and promoting pro-environmental behavioral intentions and actions. Nonetheless, a detailed exploration into the precise mechanisms facilitating these impacts remains scarce. Leveraging theories of motivation, cognition, affect, and behavior, this paper outlines four hypothesized mechanisms of influence and introduces an Embodied-Enactive Cognition Model as a novel perspective. It suggests that future research should expand its inquiry into the multifaceted factors that influence pro-environmental decision-making, deepen the comprehension of the intrinsic mechanisms at play, pioneer novel research methodologies, and diversify the array of categories and contextual applications of environmental serious games.

## 1 Introduction

The genesis of environmental issues is intricately linked to human activities and lifestyle choices, which have resulted in the unsustainable exploitation of Earth’s resources and significant disruptions to ecological balance (Abrahamse et al., 2005; Morganti et al., 2017). Addressing these critical challenges requires significant changes in human behavior, particularly in fostering pro-environmental decision-making—characterized by active concern for environmental challenges and the corresponding adoption of positive attitudes and actions (Zhang and Kang, 2016). While the importance of promoting such behaviors is well recognized, especially in both academic and industrial communities, the processes through which pro-environmental decision-making occurs remain underexplored.

This study seeks to address this gap by examining the role of environmental serious games, an innovative intervention tool increasingly utilized across various domains such as education, health, consumer behavior, and psychology (Krath et al., 2021). Existing studies confirming their effectiveness in promoting environmental knowledge, increasing awareness of environmental issues, and fostering pro-environmental behavioral intentions and actions (e.g., Zhou et al., 2019; Zhang et al., 2020; Ashfaq et al., 2021; Mi et al., 2021; Du et al., 2022). However, the topic of how environmental serious games influence pro-environmental decision-making is still only sporadically covered in empirical studies from various perspectives. There is a significant lack of theoretical reviews, particularly those exploring the underlying mechanisms of these effects.

To bridge this gap, this paper draws on theories of motivation, cognition, affect, and behavior to summarize four hypothesized mechanisms of influence that explain how environmental serious games may impact pro-environmental decision-making. Additionally, the study introduces an Embodied-Enactive Cognition Model as a novel framework for understanding these processes, offering a deeper theoretical insight into how such games can effectively foster pro-environmental decision-making. By addressing the theoretical and empirical gaps in current research, this paper not only provides new perspectives on the role of environmental serious games in influencing pro-environmental decision-making but also identifies existing challenges and offers insights and recommendations for future research and practical interventions aimed at encouraging sustainable behaviors.

## 2 Concept and current research on environmental serious games

### 2.1 Relevant concepts

Games are defined as purposeful activities or behaviors that individuals willingly engage in within specific temporal and spatial boundaries, governed by predefined rules (Suits, 1967; Zhou, 1996). Traditionally, games have been understood as forms of play that are confined to certain spaces, such as sports fields or board game tables, where participants follow established rules to achieve specific goals.

The rapid advancement of technology, particularly in video and online gaming, has expanded the traditional boundaries of games, leading to the emergence of gamification and serious games. Gamification involves applying game mechanics and design principles in non-game contexts to engage users and influence their behavior (Zainuddin et al., 2020). This concept extends beyond traditional gaming environments, incorporating elements such as points, leaderboards, and achievements into activities like education, marketing, and health care to drive user engagement and motivation (Kapp et al., 2014).

Serious games, on the other hand, are designed with a primary purpose beyond entertainment. While they still retain engaging and entertaining elements, their main goal is to address real-world challenges, often in educational, training, or therapeutic contexts (Ritterfeld et al., 2009; Yang, 2015). Unlike gamification, which applies game-like experiences to non-game settings, serious games are full-fledged games that integrate educational or functional content within an interactive and immersive gaming experience (Seaborn and Fels, 2015; Fleming et al., 2017).

Chinese scholars have further classified serious games into two categories: “strong” serious games and “weak” serious games (Li and Sun, 2019). Specifically, “strong serious games” are those that place a greater emphasis on functionality and are designed to directly address real-world problems. The scenarios created in these games closely resemble reality, enabling players to acquire professional knowledge and enhance their skills, which can then be directly applied in practical contexts. Currently, the primary business model for strong serious games is B2B, with key buyers being specialized institutions such as military units, banks, and schools. Examples of strong serious games include those used for military simulation training and medical experimentation. In contrast, “weak serious games” lean more toward entertainment while still incorporating functional elements that indirectly address real-world issues. The functionality of these games is relatively weaker, aiming to subtly impart knowledge or cultivate emotions. The virtual environments in weak serious games are less connected to reality and mainly help players improve general knowledge. In recent years, weak serious games have gained popularity, and their business model has expanded from B2B to include B2C, where they are directly marketed to the general public.

### 2.2 Overview of current research on environmental serious games

**Figure 1:**
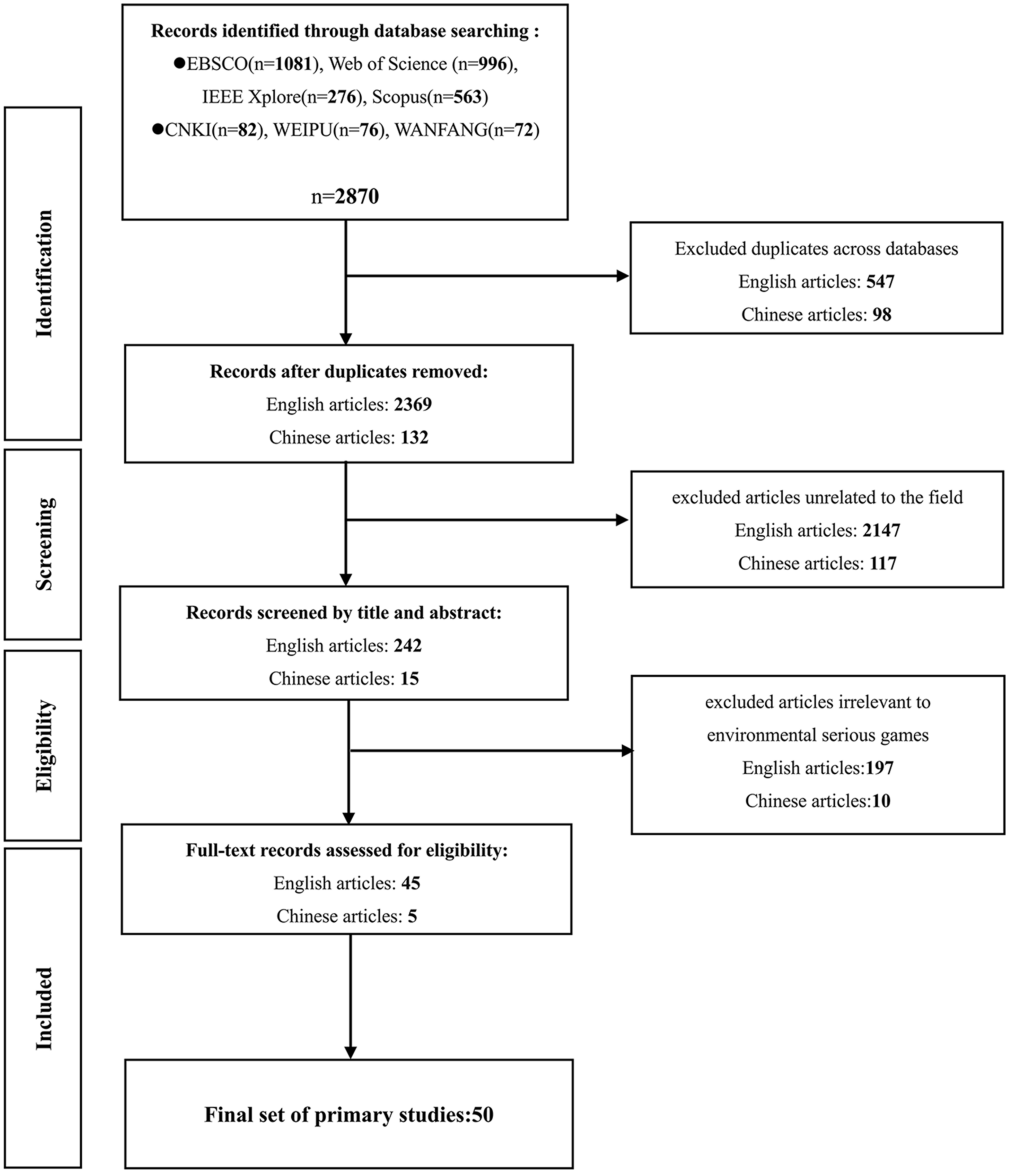
Flow diagram for study selection.

This study adheres to the widely adopted Preferred Reporting Items for Systematic Reviews and Meta-Analysis (PRISMA) guidelines (Moher et al., 2009) in order to search for empirical research literature on serious environmental games. This rigorous approach is structured into stages of identification, screening, eligibility, and inclusion, meticulously detailing the method for sourcing empirical literature pertaining to environmental serious games as depicted in Figure 1.

#### 2.2.1 Identification

The search involved using keywords such as “functional games,” “serious games,” “gamification,” “environmental protection,” “pro-environmental,” and “energy saving” in Chinese databases. For English articles, databases such as Web of Science and Scopus were utilized with search terms including (“Computer Game” OR “Digital Game” OR “Gaming” OR “Gamification” OR “Serious Game” OR “Video Game” OR “Applied Game”) AND (“energy saving” OR “pro-environmental behavior” OR “ecological footprint” OR “carbon emissions” OR “eco-feedback technology” OR “climate change”). The selection of these keywords was partly based on the researchers’ familiarity with the field and also derived from keywords used in related literature.

#### 2.2.2 Screening

The initial search yielded 2,916 English-language articles and 230 Chinese-language articles. Duplicates were removed from the CNKI, WEIPU and WANFANG databases for Chinese articles, as well as from the EBSCO, Web of Science, IEEE Xplore and Scopus databases for English articles. To achieve comprehensive coverage and avoid missing relevant studies, a manual search was also conducted on the reference lists of related thematic reviews. After removing duplicates across databases, the final tally was 2,369 English-language articles and 132 Chinese-language articles.

#### 2.2.3 Eligibility

In the second phase of screening, titles and abstracts of the retrieved literature were scrutinized, resulting in the exclusion of 2,264 articles. Subsequently, 242 English-language articles and 15 Chinese-language articles remained, requiring full-text assessments to determine their eligibility.

#### 2.2.4 Included

After conducting a full-text review, articles unrelated to environmental serious games were excluded, only 5 relevant Chinese articles and 45 relevant English articles were identified. This indicates that research in the field of serious environmental games is burgeoning and urgently requires attention.

Regarding the research themes and content, existing studies exhibit the following characteristics. Firstly, the topics of environmental serious games are diverse, including water resource management, plastic pollution, green travel, recycling, energy conservation, environmental education, and policy participation. Secondly, the forms of environmental serious games vary, including mobile applications, card games, computer games, role-playing games, and tabletop games. Third, the elements involved in environmental serious games are diverse, typically integrating multiple elements into a single game (Ouariachi et al., 2020). These elements include feedback, challenges, sharing, rewards, competition, points, badges, and avatars. Among them, challenges, feedback, competition, and social sharing are considered the most conducive to promoting players’ environmental awareness and pro-environmental behavior (Johnson et al., 2017). Unfortunately, few studies focus on the impact of different game elements on players’ pro-environmental decision-making.

In terms of research design and data analysis methods, existing studies exhibit the following deficiencies: Firstly, most studies employ self-report and interview methods to assess the behaviors or intentions of game users, which makes it challenging to eliminate social desirability bias. Only a few studies have employed mixed research methods, such as integrating behavioral intentions and actual behaviors, subjective data, and objective data (e.g., Mulcahy et al., 2020; Oppong-Tawiah et al., 2020; Whittaker et al., 2021). Secondly, there is a lack of longitudinal studies; the vast majority of existing studies solely focus on the immediate effects of serious environmental games on specific elements of players’ pro-environmental decision-making (such as cognition and behavior), without evaluating long-term effects, and with only a few studies lasting more than a year (e.g., Csoknyai et al., 2019; Wemyss et al., 2019). Thirdly, the research subjects primarily consist of young individuals, with typically small sample sizes (ranging from 10 to 600, mostly below 200), and a few studies even neglect to mention the participant count. Refer to Table 1 for details information on all papers included in the comprehensive review.

**Table 1:** Included paper details.

Research	Game name	Research method	Sample size	Environmental field involved	The main finding
Aguiar-Castillo et al. (2019)	WasteApp	Survey Research/Statistical Analysis	*n* = 141	Waste management	Can enhance tourist recycling behavior and improve a destination’s reputation.
Agusdinata et al. (2024)	HomeRUN	Survey Research/Statistical Analysis	*n* = 157	Resource conservation	The targeted social comparison messages effectively reduce carbon emissions in food and energy consumption while also prompting specific actions.
Allcott and Rogers (2014)	Opwer	Statistical Analysis	*n* = 6,000	Energy conservation	The social comparison-based energy reports trigger initial behavior cycles that diminish over time, have lasting effects even after discontinuation, and continue to influence consumers for years
Armstrong et al. (2021)	Flipping Lakes	Survey Research	*n* = 12	Environmental protection education	Flipping Lakes can lower communication barriers and increase understanding of difficult water quality concepts.
Ashfaq et al. (2021)	the Ant Forest	Survey Research/Statistical Analysis	*n* = 293	Carbon emission reduction	the user values significantly influence “reasons for” (RF), “reasons against” (RA), and attitude toward Ant Forest. Similarly, both RF (environmental benefits, social influence, hedonic motivation, and convenience) and RA (privacy concerns, usage barrier, and green skepticism) affect attitude and intention to continue using Ant Forest.
Ashfaq et al. (2022)	The Ant Forest	Survey Research/Statistical Analysis	*n* = 337	Carbon emission reduction	The study explores the influence of user experience (cognitive experience and affective experience), personal attributes (affection and altruism) and motivational factors in game play (reward for activities and self-promotion) on the continuation intention toward Ant Forest.
Barcena-Vazquez et al. (2023)	Reto Globa	Survey Research	*n* = 30	Climate change	the relevance of the use of interactive technologies to reinforce the process of raising awareness among potential museum attendees about the global warming phenomenon.
Bardhan et al. (2015)	Trash War	Survey Research/Statistical Analysis	*n* = 70	Waste management	The study proposes using persuasive technologies to encourage pro-environmental behavior and initiate waste segregation at the source.
Boncu et al. (2023)	MyEarth	Behavioral Experiments	*n* = 361	Carbon emission reduction	Using the mobile app for 8 weeks significantly improves the levels of PEB in the intervention group compared to the control group. None of the proposed interactions showed significant moderator effects.
Cellina et al. (2019)	Go Eco	Statistical Analysis	*n* = 212	Green trevel	The study concludes that the GoEco! app effectively reduced CO_2_ emissions and energy consumption in car-dependent areas like Canton Ticino but had no significant impact in regions with strong public transportation, like Zurich. It also highlights challenges in mobility trials and offers recommendations for future research.
Cellina et al. (2024)	enCompass	Behavioral Experiments	*n* = 55	Energy conservation	persuasive app use into perspective: taken in isolation, persuasive apps may exhibit limitations regarding long-lasting effects.
Chen et al. (2023)	The Ant Forest	grounded theory/Survey Research	*n* = 486	Carbon emission reduction	Results show that gamification positively influences users’ value perceptions, which then enhance environmental concerns. Value perceptions and environmental concerns mediate the relationship between gamification affordances and green consumption behaviors.
Cheng et al. (2019)	Water Ark	Survey Research/Interviews	*n* = 21	Environmental protection education	participants gradually changed from profit-oriented self-interest strategies to altruistic strategies based on social public benefit.
Csoknyai et al. (2019)	Apolis Planeta	Survey Research, Statistical Analysis	*n* = 157	Energy conservation	Serious games combined with smart metering can effectively analyze and influence energy consumption habits in residential buildings.
De Kraker et al. (2021)	Sustainable Delta	Statistical Analysis/Interviews	*n* = 150	Water resource management	The game successfully converges perspectives among participants in most sessions
Dong et al. (2023)	The Ant Forest	Survey Research/Behavioral Experiments	*n* = 666	Carbon emission reduction	Interactions within the game boost platform intimacy and love for nature, positively influencing pro-environmental behaviors, especially among those perceiving strong network externalities.
Du et al. (2020)	The Ant Forest	Survey Research	*n* = 307	Carbon emission reduction	Satisfying users’ needs for green effectiveness, enjoyment, and social gain predicts their continued use of gamified systems, with features like autonomy, achievement visibility, competition, and interactivity impacting satisfaction.
Du et al. (2022)	The Ant Forest	Netnography	Consumer comments/Tieba posts	Carbon emission reduction	Gamification elements on platforms drive green consumption behaviors through hedonic, gain, and normative goals, with short-term effects driven by hedonic and gain goals and long-term effects integrating all three goals.
Essokolo and Robinot (2022)	Plasticity	Interviews	*n* = 12	Plastic pollution	Immersive media can reduce the psychological distance associated with environmental issues such as plastic pollution.
Fijnheer et al. (2019)	Powersaver	Survey Research, Statistical Analysis	*n* = 15	Energy conservation	The Powersaver Game significantly increased energy conservation knowledge and achieved over 33% more energy savings than the control, but did not significantly change attitudes or engagement.
Fisher et al. (2021)	Animal Crossing: New Horizons	/	/	Sustainable development	Promotes pro-conservation behaviors, attitudes, and knowledge among its international players, with potential real-world conservation impacts.
Fox et al. (2020)	Recovery Rapids	Survey Research/Statistical Analysis	*n* = 190	Environmental protection education	Games can boost environmental behavior and policy support by reducing psychological distance and increasing self-efficacy through interactivity.
Gao et al. (2021)	Yudai Trench	Survey Research/Interviews	*n* = 108	Environmental protection education	Game improved knowledge on balancing urban development and disaster risk reduction, influencing decision-making and highlighting factors like economic levels and policy incentives.
Garcia-Vazquez et al. (2021)	For A Good Selfie	Survey Research/Statistical Analysis	*n* = 537	Environmental protection education	The role-play intervention significantly increased students’ intentions to refuse to buy and recycle mobile phones, with greater impact on engineering students compared to social sciences students.
Geelen et al. (2012)	Energy Battle	Survey Research/Interviews	*n* = 20	Energy conservation	Game significantly reduced energy consumption by an average of 24%, with a peak of 45% savings, and while initial energy use increased after the game, consumption remained below pre-game levels
Guo et al. (2023)	The Ant Forest	Survey Research	*n* = 504	Carbon emission reduction	Continuous use of “Ant Forest” significantly boosts offline green consumption, with behavioral reasoning theory explaining its mechanism; individuals with high environmental self-identification are more likely to engage in offline green consumption.
Gustafsson et al. (2010)	Power Agent	Statistical Analysis/Interviews	*n* = 6	Energy conservation	The Power Agent game effectively motivated teenagers and their families to alter their daily energy consumption patterns during the trial
Ho et al. (2022)	Animal Crossing: New Horizon	Survey Research	*n* = 640	Environment protection education	Positively correlates players’ perceptions of resource limits with their in-game behaviors, such as planting and resource exploitation.
Iria et al. (2020)	GReSBAS	Statistical Analysis	*n* = 400	Energy conservation	Incorporating various dashboards for information, competition, and messaging, successfully promoted energy-efficient behaviors in office buildings, resulting in a 20% reduction in electricity consumption.
Khatib et al. (2021)	Daysam	Behavioral Experiments	*n* = 47	Environmental protection education	The proposed game has increased preschooler’s awareness of renewable energy.
Khelifa and Mahdjoub (2021)	EcoDragons	/	/	Environmental protection education	It integrates ecological principles, conservation strategies, and life history concepts to engage players in ecology and conservation, promoting environmental awareness and advocacy.”
Khoury et al. (2023)	NEXTGEN	/	/	Water resource management	The game helps address the challenge of sensitizing the public and professionals to water-related issues amid climate change and resource scarcity.
Kjeldskov et al. (2012)	Power Advisor	Interviews	*n* = 10	Energy conservation	The system demonstrated potential in promoting electricity conservation by offering personalized, real-time insights into energy consumption via mobile devices.
Knol and Vries (2011)	EnerCities	Survey Research/Statistical Analysis	*n* = 653	Energy conservation	The game effectively increased their awareness and fostered more positive attitudes toward energy conservation behaviors.
Liao and Chen (2021)	the Ant Forest	Survey Research/Statistical Analysis	*n* = 420	Carbon emission reduction	Gamified interaction enhances users’ environmental awareness, which promotes pro-environmental behaviors. Environmental awareness mediates this relationship, while personal norms moderate the effect of awareness on behaviors.
Meya and Eisenack (2018)	Keep Cool	Survey Research/Statistical Analysis	*n* = 235	Environmental protection education	Simulation games can facilitate experiential learning about the difficulties of international climate politics and thereby complement both conventional communication and teaching methods.
Mulcahy et al. (2020)	Temperature Defender/Power Raid/Fully Loaded	Survey Research, Statistical Analysis	*n* = 326	Energy conservation	Used by energy companies, the app effectively promoted sustainable energy consumption, leading to increased energy-saving behaviors, positive word-of-mouth, and significant monetary savings for customers compared to a control group.
Nguyen et al. (2024)	SuDSbury	Survey Research	*n* = 14	Water resource management	Increases knowledge, comprehension, and personal norms regarding sustainable drainage solutions (SuDS) among urban residents.
O’Garra et al. (2021)	EcoChains: Arctic Crisis	Interviews	*n* = 41	Environmental protection education	Both the educational card game and the illustrated article effectively enhanced participants’ understanding of the Arctic social-ecological system. Games are valuable tools for developing systems thinking in environmental education.
Oppong-Tawiah et al. (2020)	My Backyard Garden	Statistical Analysis/Interviews	*n* = 137	Energy conservation	The results of the design cycles built on each other, demonstrating that the system decreases employees’ electricity consumption and increases their motivation to continue engaging in pro-environmental behaviors.
Orland et al. (2014)	Energy Chickens	Survey Research	*n* = 42	Energy conservation	The game reduced office plug-load energy consumption by 13% overall, with 23% on non-workdays and 7% on workdays. Participants reported greater energy consciousness and some changes in energy use outside the office.
Qin and Tian (2021)	the Ant Forest	Survey Research/Statistical Analysis	*n* = 1,246	Carbon emission reduction	Compared to “never played” individuals, both “currently playing” and “former players” show greater willingness for pro-environmental behaviors. In “currently playing” individuals, game enjoyment and competition mediate this relationship, whereas in “former players,” they inhibit it.
She et al. (2023)	The Ant Forest	Survey Research/Statistical Analysis	*n* = 228	Carbon emission reduction	Green energy collection behavior had a significant positive impact on purchase intention and pro-environmental donation. Environmental self-identity mediated the relationship between green energy collection behavior and support intention. Psychological ownership moderated the impact of environmental self–identity on ant forest support intention.
Sillanpää et al. (2024)	Minions of Disruptions	Interview and survey	*n* = 166	Climate change adaptation	The game reduced psychological distancing from climate change and enhanced participants’ sense of agency.
Van-Beek et al. (2022)	Game Name Not Mentioned	Survey Research	*n* = 49	Environmental protection education	The game effectively reduced the psychological distance of climate tipping points, making them feel more immediate and tangible to participants.
Veronica and Calvano (2020)	SeAdventure	Statistical Analysis	*n* = 46	Sustainable development	The game enhanced ocean literacy and raised children’s awareness about marine litter and biodiversity.
Wang et al. (2021)	Pear	Survey Research	*n* = 37	Sustainable development	The study found that the serious game PEAR, using geolocation and augmented reality, significantly improved participants’ knowledge and attitudes on climate change and sustainability.
Wemyss et al. (2019)	Social Power	Survey Research/Statistical Analysis	*n* = 82	Energy conservation	The intervention significantly reduced electricity use during the study, but savings were not sustained after 1 year.
Whittaker et al. (2021)	Reduce Your Juice	Survey Research, Statistical Analysis	*n* = 387	Energy conservation	A gamified app can enhance sustainable energy behavior by increasing user engagement and perceptions of value through flow experiences.
Zhang et al. (2022)	The Ant Forest	Survey Research/Statistical Analysis	*n* = 596	Carbon emission reduction	Users’ perceptions of Ant Forest’s hedonic, social, altruistic, and biospheric values positively affect their attitudes toward the app. Altruistic and biospheric values also enhance pro-environmental behavioral intentions. Positive attitudes increase gameplay continuation, which further boosts pro-environmental behaviors.

## 3 The role of environmental serious games in promoting pro-environmental decision-making

Based on existing literature, the impact of environmental serious games on players’ pro-environmental decision-making is primarily evident in three aspects.

### 3.1 Enhancing environmental knowledge and skills

Environmental serious games simplify complex knowledge into an accessible “language” (Khelifa and Mahdjoub, 2021), aiding in enhancing players’ environmental knowledge and skills and fostering an understanding of environmental conservation (Wang et al., 2021; Chen et al., 2023). Specific effects include increasing environmental knowledge, understanding energy consumption behaviors, and enhancing perception and coping abilities regarding environmental risks (Morganti et al., 2017; Douglas and Brauer, 2021; Khoury et al., 2023).

Firstly, participation in environmental serious games can increase the reserve of environmental knowledge. Games such as waste sorting, water resource management, and agricultural ecology enrich participants’ understanding and knowledge in these areas (Jouan et al., 2020; Armstrong et al., 2021). Secondly, games with energy-related themes can enhance players’ awareness of energy devices and improve efficiency, promoting recognition and understanding of energy-saving behaviors (Knol and Vries, 2011; Khatib et al., 2021). Thirdly, research on serious games with climate and environmental disaster themes indicates that engagement in these games can enhance players’ perception of environmental risks and their ability to cope with complex environmental changes (Fox et al., 2020; Gao et al., 2021).

In general, existing research consistently demonstrates the positive impact of participating in environmental serious games on players’ relevant knowledge and skills. However, there are notable shortcomings: Firstly, knowledge and skills are concepts with rich connotations and levels. Yet, most relevant studies only engage in descriptive analysis or empirical summaries, lacking in-depth exploration of the extent, scope, and specific mechanisms of the effects while also lacking standardized and unified measurement criteria. Secondly, many studies involve samples with high levels of knowledge neglecting this baseline issue which makes it difficult to exclude the additional impact of the variable of existing knowledge level. Lastly, most studies have short durations and insufficient time spans potentially leading to short-term and unstable research results that may significantly discount the applied value of these studies.

### 3.2 Boosting environmental awareness

Environmental awareness encompasses a complex psychological structure that includes cognitive elements such as values, concern for the environment, and issue awareness, as well as experiential factors arising from both indirect and direct encounters with environmental issues (Sarrica et al., 2016). Engaging with environmental serious games has the potential to bridge knowledge gaps and break down perceptual barriers, thereby enhancing public consciousness about environmental protection and sustainable practices (Orland et al., 2014; Rai and Beck, 2017).

Environmental serious games can enhance users environmental awareness by providing intuitive data. For instance, Kjeldskov et al.’s (2012) research demonstrates that these games offer intuitive data on energy usage levels to improve users’ environmental awareness. Additionally, environmental serious games can enhance environmental awareness through scenario simulations and quantified rewards, as evidenced by recent empirical research. For example, simulating animal habitats and allowing players to role-play as endangered animals have increased players’ awareness of biodiversity and environmental conservation (Veronica and Calvano, 2020; Fisher et al., 2021). Lastly, environmental serious games also play a role in spreading environmental awareness. For instance, these games can help farmer players realize the importance of soil analysis and improving fertilizer use, while disseminating this knowledge to enhance everyone’s awareness of soil environmental protection (Pruksakorn et al., 2018).

However, it is crucial to acknowledge the limitations of virtual scenarios in fully replicating real-world conditions. Virtual environments may inadvertently lead to misconceptions among players, blurring the lines between in-game actions and real-life environmental behavior. Moreover, to enhance gameplay enjoyment, developers might oversimplify or inaccurately represent environmental information. Lastly, immediate post-game evaluations may not accurately capture long-term psychological shifts among participants.

### 3.3 Encouraging pro-environmental behavioral intentions and actions

Evidence suggests that participation in environmental serious games can motivate actions conducive to environmental well-being, such as energy conservation, water saving, recycling, and pollution mitigation (e.g., Senbel et al., 2014; Reeves et al., 2015; Gangolells et al., 2021; She et al., 2023). For instance, these games can reduce the psychological distance to issues like pollution, fostering a sense of urgency and willingness to engage in behaviors that mitigate plastic pollution (Essokolo and Robinot, 2022). Moreover, the incorporation of serious game elements into household appliances has been proven to effectively stimulate energy-saving actions while significantly enhancing intentions toward sustainable behavior (Csoknyai et al., 2019). In addition to individual and household settings, environmental serious games have also shown effectiveness in reducing workplace energy consumption by incorporating gaming mechanics that track and incentivize efficient energy use among employees (Iria et al., 2020). However, some studies highlight the temporary nature of such behavioral changes, noting a tendency for energy usage to rebound post-intervention, which poses challenges for sustaining long-term conservation behaviors (Geelen et al., 2012; Allcott and Rogers, 2014; Wemyss et al., 2019).

Overall, the effectiveness of environmental serious games in enhancing pro-environmental intentions and decisions has been established; however, existing research exhibits notable limitations. Firstly, many studies are limited by homogenous and small sample sizes. Secondly, these studies predominantly focus on short-term educational impacts of environmental serious games, with minimal utilization of longitudinal methods to investigate changes over time, thereby lacking robust evidence for their long-term effects. Thirdly, research often targets demographically similar participants while overlooking potential variability in game impacts across different groups, thus reducing the applicability and value of the findings. Lastly, the immersive experiences provided by virtual games employed in this research may induce detachment from reality among participants and potentially lead to rebound or moral licensing effects.

## 4 Mechanisms of influence of environmental serious games on pro-environmental decision-making

Despite the existence of empirical evidence supporting the positive impact of environmental serious games on pro-environmental decision-making, ongoing debates persist regarding the underlying mechanisms. This paper aims to consolidate insights from a diverse range of studies in order to propose four hypotheses grounded in various theoretical perspectives.

### 4.1 Hypothesized mechanisms of influence based on motivational orientation theory

The Motivation-oriented Theory posits that the psychological impact of environmental serious games on pro-environmental decision-making is primarily driven by the dynamics of motivation formation and its determinants. The relevant research within this theory mainly includes Self-determination Theory and Goal-framing Theory. Self-determination Theory emphasizes three fundamental human needs: autonomy, competence, and relatedness (Ryan and Deci, 2017). Game elements such as badges, trophies, and collaborative interactions are strategically designed to fulfill these needs, thereby fostering intrinsic motivation toward pro-environmental actions.

Goal-framing Theory further enriches Motivational Orientation Theory by suggesting that individuals are motivated to engage in pro-environmental behaviors when they achieve three key goals: hedonic (deriving pleasure from pro-environmental decision-making), gain (obtaining benefits or saving expenses through pro-environmental decision-making), and normative (receiving social recognition for pro-environmental decision-making) (Qin and Tian, 2021). For example, users of Alipay’s “Ant Forest” can accumulate “green energy” by participating in low-carbon consumption activities. Once a certain amount is collected, the platform plants real trees on behalf of the users and issues certificates in their name. A detailed study of this game’s users revealed that the gamified platform effectively showcases multi-dimensional gamification elements, thereby meeting users’ needs and activating hedonic, gain-oriented, and normative motivations, which subsequently drive a range of short-term and long-term pro-environmental behaviors (Du et al., 2022).

This hypothesis, which is based on individual internal factors, provides some insight into the psychological mechanisms through which environmental serious games influence players’ pro-environmental decisions. However, by solely focusing on a single internal factor and overlooking the diversity of individual needs, the complexity of decision-making processes, and the impact of a varied external environment, its explanation of the influencing mechanisms tends to be somewhat limited and one-sided.

### 4.2 Hypothesized mechanisms of influence based on cognitive orientation theory

In explaining the psychological mechanisms of the influence of environmental serious games on pro-environmental decision-making, Cognitive-oriented Theory mainly includes Construal Level Theory, Self-Efficacy Theory, and Social Comparison Theory. The Construal Level Theory suggests that individuals have different levels of construal for psychological representations of objects depending on the psychological distance between their perception and cognition, which will affect individuals’ judgments and decisions (Trope and Liberman, 2010). Existing research has shown that the immersion, realism, and experiential feelings generated by games can reduce the aforementioned psychological distance, increase players’ perception of environmental risks and threats, prompt them to consider current environmental issues, and thereby encourage more pro-environmental decision-making behaviors (O’Garra et al., 2021; Sillanpää et al., 2024). For example, a climate governance-themed role-playing game examined its influence on players’ awareness of climate change-related risks from various aspects such as attention to climate tipping points, perceived severity, perceived distance, and perceived likelihood. The results indicated that participation in the game shortened players’ psychological distance perception of climate tipping points while also influencing their perceptions of geographical, social, and temporal distances. Additionally, “realism,” “proximity,” and “relevance” enhanced by the game fully aroused players’ concern for environmental issues (Van-Beek et al., 2022). Thus it can be seen that while the explanatory mechanism hypothesis of Construal Level Theory partially reveals the psychological mechanisms behind decision-making processes. However, solely applying this hypothesis to explain the cognitive and behavioral changes of players’ pro-environmental decisions in different situations cannot fully cover all phenomena and processes, while also inadequately considering the effects of social factors that may occur.

Self-efficacy Theory suggests that an individual’s behavior is influenced by their cognitive assessments of their capabilities and the expected outcomes of their actions. Self-efficacy, defined as an individual’s evaluation of their ability to execute a specific behavior (Bandura and Watts, 1996). Acts as a powerful regulatory factor, it affects behavior through intermediary processes such as selection, cognition, motivation, and psychophysical reactions. Research has demonstrated that in role-playing serious games, when players engage in activities like trash cleanup, it not only improves the environment but also enhances their self-efficacy in making pro-environmental decisions (Fox et al., 2020). This highlights the substantial explanatory power of the influence mechanism hypothesis of Self-efficacy Theory. However, while the positive effects of self-efficacy on pro-environmental decision-making are well-documented, further exploration is still needed to understand how self-efficacy impacts players, which processes are affected and what underlying psychological mechanisms are involved.

Social Comparison Theory emphasizes the role of comparing oneself to others in shaping behaviors and attitudes. In the context of environmental serious games, features such as leaderboards and rankings engage players in social comparison, enhancing their motivation for achievement and competence (Chen et al., 2020; Agusdinata et al., 2024). For example, the serious game “Social Power” with an energy theme provides users with personalized feedback on their energy usage, incorporating social comparison elements to encourage them to reduce consumption. Prolonged engagement with the game also cultivates a sense of cost-effectiveness among users (Wemyss et al., 2019). Studies indicate that integrating social comparison and outcome evaluation elements into serious games can influence cognitive factors and actual behaviors in pro-environmental decision-making, thereby validating the hypothesis of the influence mechanism of social comparison theory. However, this hypothesis does not thoroughly analyze the characteristics of those who are comparing and being compared as individual comparisons can vary significantly; it also lacks monitoring of the psychological and feedback processes involved in these comparisons. Additionally, both the hypothesis and associated studies do not adequately address the nature of social comparisons themselves, such as whether they are intentional or unintentional, overt or covert, upward (comparing with those who perform better) or downward (comparing with those who perform worse), leading to limitations in revealing the mechanisms of influence.

### 4.3 Hypothesized mechanisms of influence based on emotional orientation theory

Emotion-oriented theories emphasize how players’ emotions during gameplay can impact their subsequent decisions and behaviors. Key emotional theories in this research include Feelings-as-information Theory and Empathy Theory. The Feelings-as-information theory considers various subjective experiences, such as moods, emotions, metacognitive feelings, and physical sensations—as sources of information that impact human decision-making. Individuals often use their feelings as inputs for making decisions, with different emotions providing distinct types of information (Schwarz and Clore, 1983). Based on the Feelings-as-information Theory, Whittaker et al. (2021) established three dimensions of flow experience, customer engagement, and behavioral value while studying energy-saving themed serious games. The results indicate that the flow experience generated by players in the game can enhance their perception of engagement with the game and the value of sustainable energy-saving behaviors, thereby positively influencing their willingness to engage in pro-environmental behaviors.

Empathy Theory posits that empathy is the ability of individuals to experience and understand the feelings or emotional states of others (Decety and Lamm, 2006). If individuals empathize with others or a particular environment, they will exhibit caring and friendly behavior toward the target object (Font et al., 2016). Existing research has proven that placing individuals in gaming situations can more effectively evoke empathy compared to having them imagine target situations. For instance, a study divided 537 university students into experimental and control groups to participate in a role-playing serious game. The results demonstrated that role-playing induced players’ sense of empathy, motivating them to engage in green sustainable behaviors such as refusing to buy phones and agreeing to recycle phones (Garcia-Vazquez et al., 2021).

The impact mechanism hypothesis, which is based on the Emotion-oriented Theory, offers a novel perspective for understanding how environmental serious games affect players’ pro-environmental decision-making. However, it can only explain the aforementioned effects observed in environmental serious games that emphasize emotional communication, such as the influence of emotional experiences in situational games like role-playing. It does not have explanatory power for rational pro-environmental decision-making behavior triggered by non-emotional factors. Furthermore, like the preciously mentioned hypotheses, these hypotheses also lack comprehensive and multi-faceted considerations.

### 4.4 Hypothesized mechanisms of influence based on behavioral orientation theory

Over the years, various theories describing factors that influence behavior have been widely applied in the research field of serious games, such as Behavior Reasoning theory, Theory of planned behavior, and Technology Acceptance Model. These theoretical hypotheses all consider subjective norms and attitudes as direct antecedent variables of behavioral intention, emphasizing their importance in explaining behavioral intentions and their impact on individuals’ actual behavior.

Traditional Rational Behavior Theory posits that any factor can only influence behavior through attitude and subjective norm (Ajzen and Fishbein, 1973). Westaby (2005) introduced the concept of behavioral rationality into the aforementioned theory, proposing Behavior Reasoning Theory. It claims that behavioral reasons (adoption/rejection) collectively influence pro-environmental decision-making behavior (e.g., green consumption). This theory also emphasizes that the rationality of behavior plays a bridging role among an individual’s values, attitudes, behavioral intentions, and behaviors, organically integrating them (Wang and Du, 2016; Guo et al., 2023). Ashfaq et al. (2021) conducted a study on 293 users of “Ant Forest” demonstrating that reasons for performing green consumption behavior (e.g., environmental benefits, social impact, pleasure motivation, and convenience) can enhance players’ willingness to continue using “Ant Forest” while reasons for rejecting this behavior (e.g., privacy concerns, usage barriers, and green skepticism) will lead to players avoiding green behavior.

As an extension of Rational Behavior theory, the Theory of planned behavior introduces the concept of perceived behavioral control, advocating that individuals’ positive evaluation of a behavior (attitude), belief that their peers will support the behavior (subjective norm), and perception that the behavior is within their control (perceived behavioral control), these three factors can most accurately predict their behavioral intentions (Ajzen, 1991). A recent study verified this hypothesis using the augmented reality (AR)-based serious game “PEAR.” By comparing players’ attitudes, subjective norms, perceived behavioral control, and behavioral intentions in the game and post-game, the study demonstrated that participating in the game can increase players’ awareness and relevant attitudes toward sustainable development and climate change (Wang et al., 2021).

The technology acceptance model considers perceived usefulness and perceived ease of use as determinants of behavioral attitude. A thematic serious game, designed to promote recycling based on the technology acceptance model, conducted an experimental study on 141 players. The study proved that players’ perception of the games’s usefulness and ease of use would influence their satisfaction, thereby affecting their recycling behavior (Aguiar-Castillo et al., 2019).

The impact mechanism hypothesis of Behavior-oriented Theory offers a partial explanation of the relationship between individual psychological factors and specific behaviors, incorporating some non-rational elements and thereby enriching the understanding of the underlying mechanisms. However, the hypothesis still falls short in its consideration of rational behavior assumptions, as it overlooks crucial internal factors such as emotions and will, as well as external factors like social norms and social comparison. Furthermore, the hypothesis fails to account for the possibility that players may be influenced by different factors at various stages of engagement, which is not adequately addressed in the current explanation.

**Figure 2:**
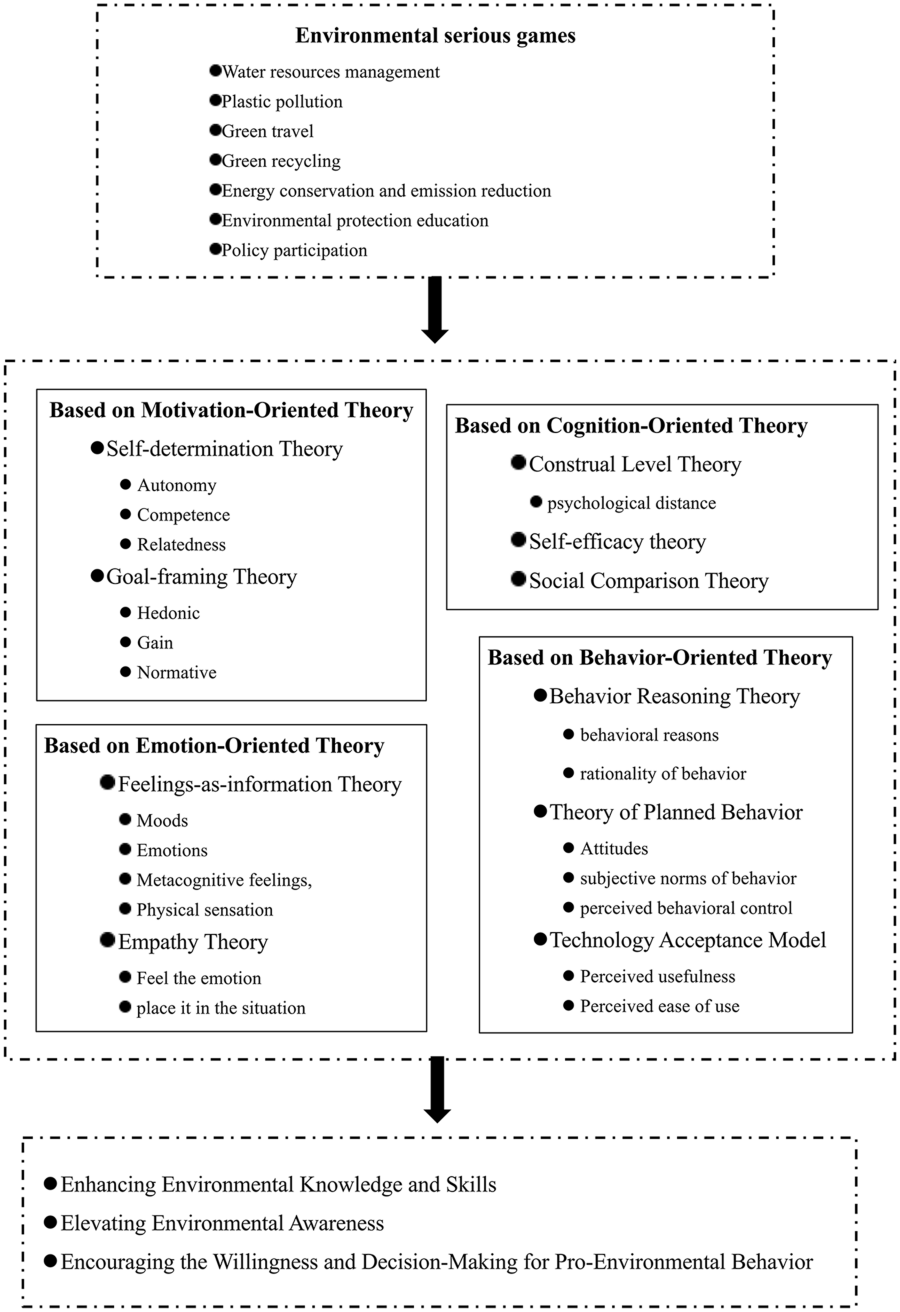
The research framework on the role of environmental serious games in pro-environmental decision-making and their mechanisms of influence.

In summary, each of the four hypotheses mentioned above has its own emphasis and provides certain explanatory power. This study summarizes the framework for research the role of environmental serious games in pro-environmental decision-making and their mechanisms of influence (see Figure 2). However, considering the complexity of the human psychological structure and function, the impact of environmental serious games on pro-environmental decisions (including environmental knowledge and skills, environmental awareness, and environmental behavior) is likely to result from the interaction of multiple factors such as motivation, emotion, behavior, and cognition. However, the aforementioned hypotheses still have significant limitations: Firstly, each hypothesis is limited to explaining all processes based on one aspect of individual behavior initiation, and does not elucidate the primary and secondary roles of various influencing factors. Secondly, each hypothesis neglects the organic integrity of individual psychology, and the fragmentation of individual psychological factors will inevitably lead to an inability to accurately reflect and fully understand specific psychological phenomena, which is detrimental to solving practical problems. Finally, some hypotheses are interrelated and overlapping, but existing research lacks discussion on the commonalities and differences of each hypothesis, as well as a comprehensive review of numerous theoretical systems, hindering the derivation of integrative theoretical concepts with universal significance from multiple hypotheses.

## 5 Discussion and conclusion

### 5.1 Theoretical contributions

The study summarizes the research framework on the role of environmental serious games in influencing pro-environmental decision-making and the mechanisms underlying this influence. While each of the four previously discussed hypotheses offers distinct insights, they are largely constructed from single-perspective approaches, limiting a comprehensive understanding of how these games impact pro-environmental behavior. To address these gaps, it is crucial to view the psychological processes involved as a holistic entity, considering the entire cycle of psychological activities within the context of serious games, as well as the interaction between the individual and the external environment.

**Figure 3:**
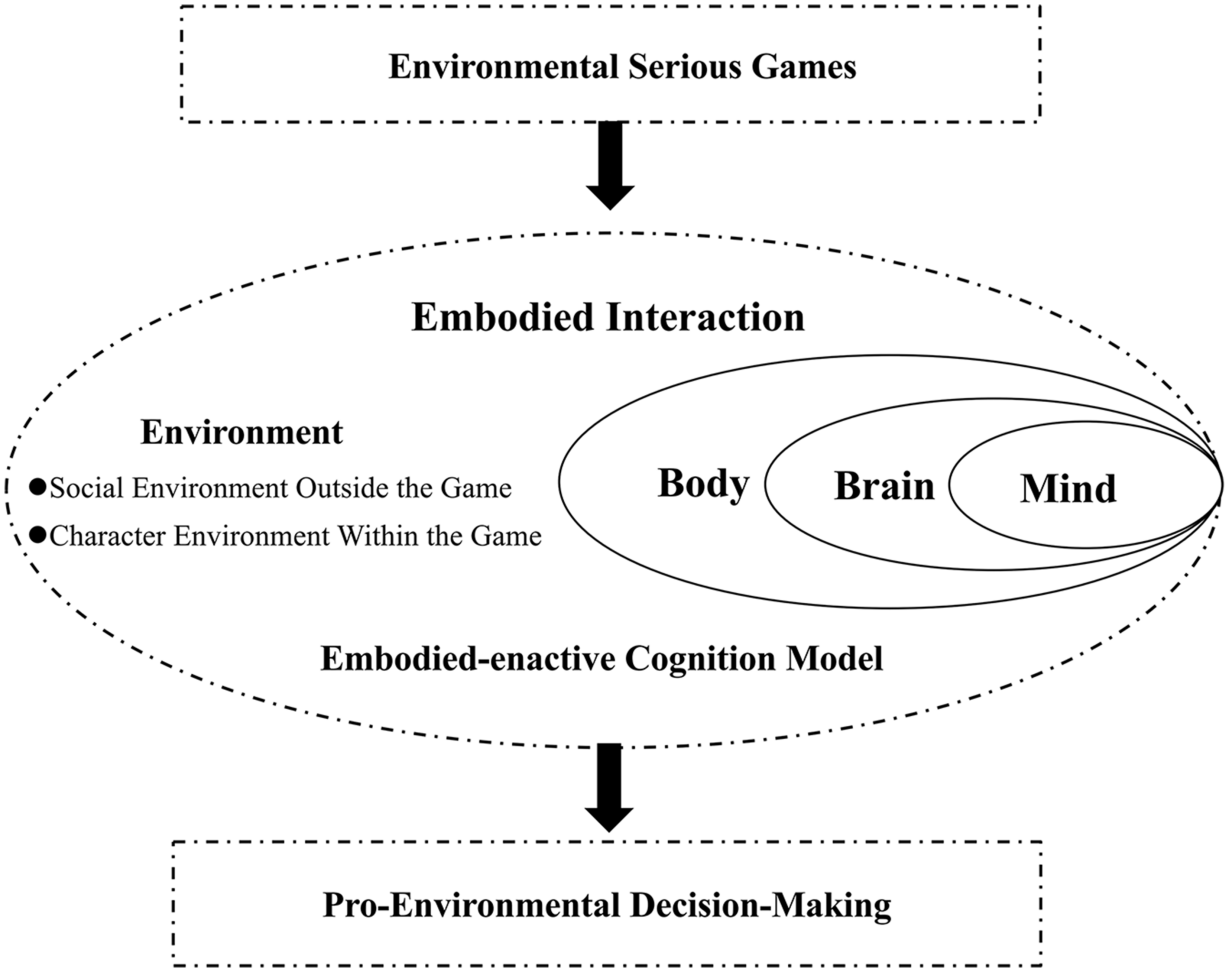
Embodied-enactive cognition model.

In response to these limitations, this paper introduces the Embodied-Enactive Cognition Model (as shown in Figure 3), grounded in Embodied cognition Theory. This model provides a more systematic and integrated theoretical framework, offering deeper insights into the mechanisms by which environmental serious games influence pro-environmental decision-making. By combining cognitive, emotional, and environmental factors into a unified system, the model moves beyond the scope of existing hypotheses, providing a novel perspective that addresses the interplay between mind, body, and environment. This model not only fills existing gaps in the literature but also opens up new directions for future research.

Embodied Cognition Theory treats individuals as unified entities, linking cognition, perception, and bodily states with their surrounding physical and social environments. This perspective posits that our understanding of the world is generated within a dynamic “perception-action” loop, where emotional and cognitive processes are deeply intertwined (Liu and Gao, 2015; Ye et al., 2021). The model’s holistic approach positions the mind, brain, body, and environment as interdependent components of a single system, thus offering new insights into the psychological mechanisms that drive pro-environmental decision-making in the context of serious games.

Moreover, the emergence of technologies like “ChatGPT” and the development of the metaverse highlight the extension of psychological processes beyond the brain, encompassing external environments and digital realms, thereby broadening the scope of interaction and influence (Su and Ye, 2023). These advancements further support the relevance and potential of the Embodied-Enactive Cognition Model in capturing the complex, multi-layered interactions that occur in environmental serious games.

To advance the application and understanding of this model, future research should explore three critical levels:

Firstly, at the micro-level, it is essential to conduct detailed analyses of the game elements, functionalities, and technical features of environmental serious games. Research should aim to uncover the intricate relationships between these characteristics and users’ perceptions, psychological processes, and resulting behaviors. Understanding how specific game elements engage users’ psychological processes and enhance their decision-making capabilities is crucial.

Secondly, at the macro-level, research should focus on the interactions between game elements and subjective factors influencing pro-environmental decisions, such as emotions, motivation, bodily perceptions, and social norms. Additionally, it is important to explore how social structures, resource distribution, and societal mechanisms shape these decisions. By identifying key factors and mechanisms that influence group-level decisions, and examining differences between individual and collective participation, the model’s explanatory power can be significantly enhanced. This comprehensive approach will deepen our understanding of how environmental serious games promote pro-environmental decision-making, thereby enriching both theoretical frameworks and practical applications.

Thirdly, the exploration of neural mechanisms underlying the effects of environmental serious games represents a promising area for further investigation. While existing studies often focus on behavioral analysis, there is a significant gap in understanding the neural processes that drive these behaviors. Recent neuroimaging research suggests a connection between pro-environmental decisions and anticipatory brain processes (Brevers et al., 2021). With the advent of technologies like the metaverse, new opportunities arise to probe these neural pathways, offering robust support for future research into brain-based networks that facilitate environmental decision-making. Integrating cognitive neuroscience with behavioral methodologies will provide a more nuanced and comprehensive understanding of how engagement with environmental serious games translates into pro-environmental behaviors.

### 5.2 Practical implications

Firstly, environmental serious games, with their unique interactive and immersive experiences, can effectively influence consumers’ environmental awareness and behavioral inclinations. By partnering with such gaming platforms, companies can actively guide consumers toward adopting sustainable consumption habits. By integrating the consumption data of green products or services with game elements, companies can create more engaging consumer scenarios, subtly instilling environmental values during enjoyable gaming experiences, and gradually internalizing these values into consumers’ everyday behaviors. This not only enhances customers’ environmental consciousness but also strengthens their loyalty to the company. Furthermore, collaboration with environmental serious games allows companies to significantly enhance their green brand image. When consumers experience the satisfaction and sense of accomplishment derived from engaging in environmentally friendly behaviors within the game, they are more likely to become loyal customers and, through word-of-mouth, attract additional potential consumers. Particularly as green consumption becomes increasingly mainstream, a company’s environmental practices will become a critical factor in consumer choice, helping the company to stand out in a competitive market.

Secondly, environmental serious games offer governments a flexible and cost-effective tool for implementing external interventions. These games not only reduce the execution and supervision costs commonly associated with traditional policy interventions but also circumvent potential ethical and legal challenges. This approach not only makes the promotion of environmental behaviors more economically and socially efficient but also allows for the participation of various societal actors in environmental governance, embodying the principle of multi-stakeholder governance. Governments can also leverage environmental serious games to overcome the challenges of collective action and cultivate green consumption habits among the public. Through gamification strategies, such as friend rankings and point systems, governments can transform broad environmental goals into manageable small-scale collective actions that can be monitored, thereby encouraging citizens to voluntarily engage in environmentally friendly behaviors in their daily lives. Additionally, governments can collaborate with internet platforms to establish a citizen green consumption behavior data center, which would record and provide real-time feedback on green consumption data, thereby enhancing public engagement and sense of achievement, ultimately fostering long-term green consumption habits.

Thirdly, for environmental social organizations, environmental serious games offer a broad-reaching platform that can effectively engage millions of users. These organizations can utilize these games as a platform to design and promote environmental public welfare activities, targeting highly active game users as the core audience. Through the use of in-game incentive mechanisms and interactive designs, social organizations can more easily attract public participation, thereby promoting environmental awareness on a larger scale.

### 5.3 Future research directions

#### 5.3.1 Exploring the nuanced factors influencing pro-environmental decision-making through environmental serious games

Although existing research has explored a range of influential factors, there are still several key variables that have not been examined. To develop more effective intervention strategies and increase the applicability of these studies, future research could enhance its explorations in the following areas:

Firstly, it is crucial to investigate the impact of demographic variables such as gender, education level, income, and sample type on pro-environmental decision-making—a critical global initiative. Despite significant evidence showing their influence on environmental behavior, most existing studies have overlooked these demographic variables. For example, a study conducted in Russia found that women exhibit stronger environmental consciousness than men, and individuals with higher levels of education are more inclined to engage in pro-environmental behaviors than those with lower education levels; however, high-income groups exhibit a need for increased environmental awareness (Sautkina et al., 2022). Similarly, research in China indicates that women tend to take more proactive environmental actions in private spheres, while men excel in public domains; additionally, awareness of gender equality positively influences pro-environmental behaviors across various sectors (Du and Zhang, 2020). Therefore, future research should explore potential gender differences in the impacts of environmental serious games on pro-environmental decisions and examine how both in-game and real-world gender roles influence these outcomes. Regarding education, while existing studies have predominantly focused on well-educated players, there is a significant research gap concerning less educated groups. Furthermore, most studies overlook high-income populations, with only a few addressing differences in income levels among players. In terms of sample types, research is often limited to convenient samples of university and school students, neglecting more representative societal samples. Future studies should expand their sample base to include a broader range of societal groups such as civil servants, entrepreneurs, various professionals, and special populations in order to develop more realistic and targeted policy recommendations.

Secondly, it is crucial to explore the impact of collective-level factors such as social norms and social identity. Environmental crises are collective phenomena, and environmental regulations and policies are typically framed as collective decisions rather than individual opinions, expressed, implemented, and evaluated through communal concern. Thus, studies on the impact of environmental serious games on pro-environmental decisions should place more emphasis on this collective dimension more. However, most existing research has focused solely on individual factors that affect pro-environmental decisions. While some studies based on social comparison theory have illustrated the intervening role of social forces, there is still a scarcity of research on how social norms and identities within environmental serious games influence pro-environmental decisions. Furthermore, substantial evidence exists that beliefs about others’ behaviors and attitudes are reliable predictors of individual environmental actions (Fritsche et al., 2017). However, there is no unified understanding of how social norms operate within the influence process of environmental serious games on pro-environmental decisions. Future studies could investigate various norms, including descriptive, injunctive, perceived, and actual norms. Additionally, the process of social identification—a key driver in environmental assessment and response—is often neglected. Examining how an individual defines their group, comparisons between groups, and how such social references influence decision-making in environmental serious game scenarios represents a promising direction for future research.

Thirdly, it is essential to conduct localized research to explore the impact of environmental serious games within the context of China’s economic and cultural landscape. International research has already established a strong link between pro-environmental decision-making and factors such as personal values, regulatory environments, and levels of economic development (Sautkina et al., 2022). China, with its vast diversity in ethnicity, economic levels, and regional cultures, offers a crucial context for in-depth exploration of these dynamics. The variations in environmental attitudes and behaviors across different stages of development highlight the potential for localized research to significantly influence the development of customized approaches to environmental challenges, especially in developing countries. Conducting such research in China could generate strategies and insights that reflect the complex interaction between cultural and economic factors, shaping environmental behaviors, and offering a blueprint for similar initiatives worldwide.

#### 5.3.2 Pioneering methodologies for studying the impact of environmental serious games on pro-environmental decision-making

Most existing research in this field has relied on self-reports and interviews to assess game users’ behaviors or intentions, approaches that inherently introduce social desirability bias. This bias tends to lead users to report more positive pro-environmental behaviors than they actually practice. Additionally, there is often a significant discrepancy between behavioral intentions and actual behaviors, potentially compromising the validity of these assessments. Future research could advance through the following innovative approaches:

Firstly, future studies should increasingly employ advanced neuroimaging technologies such as Functional Magnetic Resonance Imaging (fMRI) and Magnetoencephalography (MEG). These technologies offer more precise processing of imaging signal data and enable rigorous neuroscience experiments that monitor changes in brain networks. For instance, recent neuroscience research utilizing a cue exposure paradigm with fMRI monitoring has scanned players’ prefrontal cortex, revealing that sustainable behaviors activate a brain network consisting of the ventromedial prefrontal cortex, hippocampus, and parahippocampal gyrus (Brevers et al., 2021). Moreover, combining field tracking surveys and behavioral experiments can effectively integrate game behavior with actual pro-environmental actions—like home energy conservation, office energy saving, recycling, and low-carbon commuting—blending subjective and objective data to yield findings with greater ecological validity.

Secondly, it is crucial to assess the long-term impacts of environmental serious games on pro-environmental decision-making. While existing studies predominantly show immediate improvements in specific pro-environmental behaviors resulting from game interactions, these short-term behavioral changes may wane over time. The majority of research lacks long-term tracking of the enduring effects of environmental serious games, challenging the ability to verify if players maintain their pro-environmental behaviors over extended periods. Therefore, focusing on longitudinal observations and evaluations of user behaviors, along with exploring strategies to ensure the sustainability of qualitative changes in players’ pro-environmental decision-making, should become pivotal directions for future research efforts. This approach not only enhances the methodological rigor of studies but also significantly contributes to the development of practical and impactful environmental gaming strategies.

#### 5.3.3 Diversifying categories and application scenarios of environmental serious games

Serious games have become a significant segment within the global electronic gaming industry. However, in China, this genre is still in its infancy, characterized by a lack of well-defined categories and comprehensive academic disciplines dedicated to its study. The application range of environmental serious games, in particular, is relatively narrow. Identifying of high-quality environmental serious games and the establishment of distinct categories and standards for these games are crucial steps toward catalyzing the development of innovative and effective new offerings in this domain.

Additionally, the environmental serious games that have predominantly been the focus of research to date are often simplistic 2D games. Such games frequently fall short in terms of interactivity and entertainment value, thereby struggling to fulfill players’ experiential demands. With the advent and continuous evolution of metaverse technology, alongside the rapid progression of digital technology, games are poised to become one of the first and most influential product types within the emerging metaverse landscape (Yu, 2021; Lan, 2022; Tsinghua University Metaverse Culture Laboratory, 2023). This technological leap forward is expected to significantly influence the realm of environmental serious games by introducing a wide range of game varieties and application scenarios that promise to enrich player experience and effectively encourage pro-environmental decision-making.

Looking ahead, future research endeavors should focus on classifying environmental serious games while examining how different game types may uniquely influence pro-environmental decision-making behaviors. Special attention could be directed toward immersive experiences offered by advanced Augmented Reality (AR) and Virtual Reality (VR) games. These games not only transport players into engaging virtual environments but also facilitate interactive experiences that are much more engaging than those provided by traditional 2D games. Investigating the immersive quality of these games and their impact on fostering pro-environmental decisions offers a promising area for research. Such studies can provide insights into the comparative effectiveness of immersive versus non-immersive gaming experiences in promoting sustainable behaviors, thereby contributing valuable knowledge to the field and guiding the development of future environmental serious games.
